# Empathy and validation in chronic pain couples: a systematic review

**DOI:** 10.1097/PR9.0000000000001343

**Published:** 2025-10-21

**Authors:** Karolin Teichmüller, Hripsime Galstyan, Lisa M. Bas, Gudrun Kindl, Philipp Kanske, Heike L. Rittner, Andrea M.F. Reiter

**Affiliations:** a Department of Psychology, University of Würzburg, Würzburg, Germany; b Department of Child and Adolescent Psychiatry, Psychosomatics and Psychotherapy, Centre of Mental Health, University Hospital Würzburg, Würzburg, Germany; c Department of Anaesthesiology, Intensive Care, Emergency and Pain Medicine, Centre for Interdisciplinary Pain Medicine, University Hospital Würzburg, Würzburg, Germany; d Faculty of Psychology, Institute of Clinical Psychology and Psychotherapy, Dresden University of Technology, Dresden, Germany; e Department of Psychology, Faculty of Psychology and Educational Sciences, Babeş-Bolyai University, Cluj-Napoca, Romania

**Keywords:** Chronic pain, Noncancer pain, Invalidation, Marital interaction, Spouses

## Abstract

Chronic pain is significantly influenced by social interactions, especially within close relationships. However, it is unclear how exactly partner responses such as solicitousness or empathy, affect pain-related and relationship outcomes. In October 2024 and July 2025, we systematically searched APA PsycInfo, Web of Science, and PubMed for studies assessing the impact of empathic or validating behavior on pain-related and relationship outcomes in adult romantic couples with at least 1 chronic pain patient. Eleven studies (4 on the same sample) were included, with sample sizes between 8 and 145 couples. In most studies, participants' mean age was older than 50 years. The most common pain condition was painful knee osteoarthritis, followed by other musculoskeletal issues. The Risk of Bias Utilized for Surveys Tool revealed a moderate risk of bias in the included studies. Four studies found a positive impact of empathy on pain-related outcomes, particularly physical functionality. No study reported adverse effects of solicitousness on pain or functioning. Four studies revealed positive effects of empathy on relationship outcomes, eg, a buffering effect of empathy on the association between depressive symptoms and marital quality. A largely consistent result was the detrimental impact of invalidation. However, some studies reported nonsignificant or partially contradictory findings, especially in male patient couples. These results largely align with Intimacy and Coping Theories and highlight the beneficial function of empathy and validation in chronic pain. However, variability in the definition and measurement of empathy complicates comparisons. In clinical practice, promoting spousal empathy and reducing invalidation could enhance pain management.

## Abstract 2

Supplemental Digital Content is Available in the Text.

Spousal empathy and validation were associated with beneficial pain-related and relationship outcomes in chronic pain couples across most studies. The opposite was true for invalidation.

## 1. Introduction

Chronic pain is a debilitating condition defined as pain that persists or recurs for more than 3 months.^61^ It has a multifactorial origin,^42^ making a biopsychosocial framework necessary to understand the development and maintenance of chronic pain. This review focuses on the social aspect of this model by summarizing the current knowledge on the impact of empathic or validating behavior on pain-related and relationship outcomes in adult romantic couples. Indeed, as an individual with chronic pain (ICP) navigates his or her daily life, pain influences and is influenced by interactions with others, particularly by those closest to them, such as their significant others.^2,17^

According to the social communication model of pain,^14^ verbal or nonverbal expressions of pain inform others about the ICP's pain experience and encourage observers to provide support and care. The other's responses can, in turn, shape the ICP's pain experience. Thus, social interactions can affect both the expression and management of pain.^14^ To understand these effects, different frameworks have been applied: A classical operant model of pain^21^ suggests that pain behaviors, ie, the expression or display of pain, can either be reinforced or punished by a significant other's response, influencing the likelihood of their occurrence. Within this framework, solicitousness, ie, “the provision of instrumental support and expression of emotional concern,”^41^ is assumed to reinforce pain behavior, with potentially detrimental effects on patient functioning. Dismissive or negative partner reactions should, in turn, lead to a reduction of patient pain behavior.^32^ However, a subset of verbal pain behaviors seems to deviate from operant theory predictions, specifically emotional disclosures of pain-related distress.^10^ These verbal expressions of feelings such as anger, sadness, or worry about pain have been conceptualized as controlled and goal-directed communication,^10^ to which social interaction partners should ideally respond in an empathic or validating manner.^36^ When we validate, we communicate to another that their experiences, including their pain, are real and legitimate.^63^ Newer models suggest that validation plays a beneficial role in the coping processes for ICPs. For instance, intimacy might develop particularly in situations where one person responds in an empathic and validating manner to another person's disclosure of personal experiences.^52^ Similarly, Linehan's biosocial model^35^ proposes that empathic responding or validation soothes an individual and reduces negative emotional arousal, which might positively affect pain adjustment (ie, decreased pain intensity and behavior). In a chronic pain context, a partner's empathic or validating response to an ICP's self-disclosure of pain-related thoughts and feelings could foster affection and decrease pain intensity and pain behavior.^18^ By contrast, invalidating interactions, such as hostile or dismissive partner responses, may interfere with emotion and pain regulation.^9^

In sum, empathy emerges as an important element that can deepen intimacy, support emotion regulation, and facilitate coping efforts within relationships.^3^ However, reaching a unified definition and conceptualization of empathy has proven challenging.^51^ Narrow approaches view empathy as the sharing of the affective experience of others.^16^ Here, we adopt a broader conceptualization of empathy as a multidimensional construct including both cognitive and affective processes and their combination.^55^ Cognitive components reflect the ability to accurately infer another's psychological state^29^ or adopt their point of view (perspective-taking).^15^ Affective branches of empathy include the sharing of others' emotions or pain, empathic concern or compassion (ie, a feeling of warmth and concern for others),^50^ and empathic distress (ie, self-oriented feelings of anxiety or unease).^15^ The concept of validation involves accepting and understanding responses to others' experiences,^7^ as opposed to invalidation, which consists of disrespectful, contemptuous, or nonaccepting responses. Transferred to the chronic pain context, *pain-validation*, described as “communicating belief and acceptability of the sufferer's expressions of pain,”^43^ serves to acknowledge the sufferer's experience.

Taken together, social interactions with close others can either enhance coping and facilitate optimal adjustment or contribute to the maintenance or even exacerbation of chronic pain. In addition, partners of ICPs must engage appropriately without becoming emotionally overwhelmed. They need to maintain this balance over an extended period because of the persistent nature of chronic pain, while excessive empathy is considered a risk factor for the development of empathic distress and also mental health issues.^28^ While acute pain is a prominent model for studying empathy,^19,31,57^ the scientific literature still lacks a comprehensive discussion of empathy's impact on chronic pain. An open question is whether the assumed positive effect of empathy in romantic relationships is consistent across studies. Therefore, this systematic review addresses this gap by evaluating the impact of empathic partner responses and behaviors on pain-related outcomes for the affected partner and relationship outcomes for couples managing chronic pain. It aims to provide insights for future research directions and implications for therapeutic approaches.

## 2. Methods

This systematic review was pre-registered with PROSPERO (Registration Number CRD42024600201, https://www.crd.york.ac.uk/PROSPERO/view/CRD42024600201) and was conducted following the guidelines of the Preferred Reporting Items for Systematic Reviews and Meta-Analyses (PRISMA).^47^ Eligibility criteria were defined based on the following PICO framework: The population (P) comprised adult couples in a romantic relationship where one partner deals with a chronic noncancer pain condition. Cancer pain was excluded because of its specific characteristics and management. Mainly, noninterventional studies (I) were expected to be included, focusing on observing empathic or validating responses between partners. Nevertheless, interventional studies promoting these behaviors between partners would also have been eligible. Valid comparisons (C) could be against solicitous (overprotective, reinforcing), nonempathic or invalidating (dismissive, hostile), or neutral responses from the partner. Studies including healthy controls, ie, couples without chronic pain, would also have been eligible. However, studies without any comparison group (eg, with a purely correlative design) were also included. The outcome measures (O) were restricted to pain-related outcomes (ie, reduced pain intensity, pain-related disability, pain medication, improved pain coping strategies, and decreased pain behaviors in the affected partner) or relationship outcomes (ie, increased relationship satisfaction, emotional intimacy, and marital functioning) or both.

Only peer-reviewed and published original studies written in English or German were considered. There were no restrictions regarding publication year or study design.

### 2.1. Search strategy

**Figure 1.**
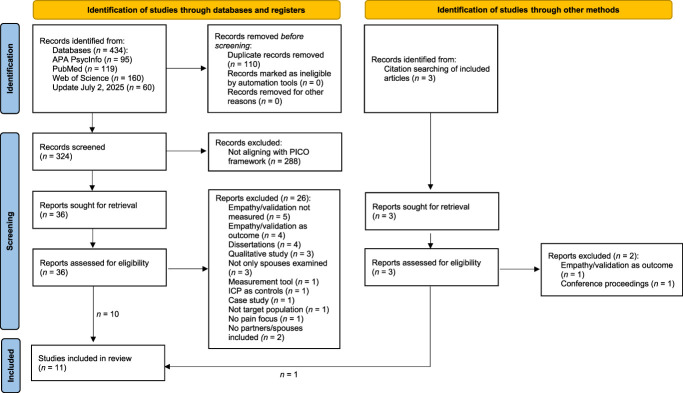
PRISMA flow diagram. Template for the creation of the diagram taken from Page et al. (2021).^47^ PRISMA, preferred reporting items for systematic reviews and meta-analyses.

A systematic literature search was conducted through 3 major electronic databases (APA PsycInfo, Web of Science, and PubMed) on October 2, 2024, using the following search terms in English and German: (“empath*” OR “validat*” OR “empathic respon*” OR “validating respon*”) AND (“chronic pain” OR “chronic illness” OR “long-term pain”) AND (“partner” OR “couple*” OR “spouse*” OR “marital relationship” OR “romantic relationship” OR “dyad*”) NOT “cancer pain.” As an additional search strategy, an ascendancy approach was used to identify further studies by screening the references of included studies (Fig. 1). The search procedure was repeated on July 2, 2025, to identify any newly eligible studies.

### 2.2. Screening process

All search results were imported into the Rayyan tool,^46^ and duplicates were removed before screening. During the first screening round, studies were screened by title and abstract. During the second round, studies were screened by full text and selected for data extraction according to our PICO framework. Owing to pragmatic reasons, only 1 reviewer (October 2024: H.G., July 2025: K.T.) was involved in the entire screening process. In case of uncertainty, H.G. and K.T. resolved ambiguity through discussion. If consensus could not be reached, input from A.M.F.R. was sought to make a final decision. The screening process was not blinded.

### 2.3. Data extraction and synthesis

Data were systematically extracted from each included study using a predesigned extraction template specific to the objectives of this review, which was slightly adjusted during the extraction process. The extracted data encompassed the following key areas: (1) study characteristics, including author names, publication year, study design, and sample size; (2) participant demographics, such as age (*M, SD*), gender distribution, and health status (if possible with a specified chronic pain condition); (3) empathy, including its conceptualization and operationalization; (4) other variables as well as the tools used for measurement; (5) results, specifically the main findings and statistical significance (ie, *P*-values, confidence intervals, and effect sizes if reported); and (6) an assessment of the study quality (see below). The extracted data are summarized in Table 1.

**Table 1:** Results of data extraction.

Study characteristics				Participant demographics				Empathy		Other variables and measurement tools	Results	QA
Authors	Year	Study design	Sample size N	Age *M* (*SD*)	Gender	Race/ethnicity	Health status/pain condition	Conceptualization	Measurement			Score (0–8)
Cano, Barterian, and Heller	2008	Observational	92 couples	ICPs: 52.05 y (13.04) Partners: 52.87 y (15.17)	ICPs: 52.7% female	ICPs: 47.3% African American, 45.2% Caucasian, 3% other, 4.3% no information Partners: 46.2% Caucasian, 45.2% African American, 2% other, 7.5% no information	Couples with at least 1 partner with chronic musculoskeletal pain: mostly back pain (50%) and osteoarthritis (24%)	Validation (=empathic response to a partner's emotional expression) vs. Invalidation (=nonempathic response)	2 × 10 min videotaped marital interaction about the impact of pain on their lives Raters coded validation and invalidation on separate global rating scales (1–7) using the Validation and Invalidation Behavior Coding System (VIBCS)	(1) Perception of spouse responses to pain: Multidimensional Pain Inventory (MPI)/Multidimensional Pain Inventory-Spouse version (MPI-S) (2) Relationship quality: Dyadic Adjustment Scale (DAS), romantic partner-specific support scale	(1) Validation by the ICP is positively correlated with marital satisfaction in both partners (*r* = 0.19, *P* < 0.05 for ICP; *r* = 0.18, *P* < 0.05 for spouse) and perceived spousal support reported by ICP (*r* = 0.20, *P* < 0.05) (2) Validation by spouse was positively correlated with marital satisfaction in both partners (*r* = 0.24, *P* < 0.05, respectively) (3) Invalidation by the ICP was negatively correlated with marital satisfaction in both partners (*r* = −0.21, *P* < 0.05 for ICP; *r* = −0.26; *P* < 0.01 for spouse) and negatively correlated with perceived spousal support reported by both partners (*r* = −0.44, *P* < 0.01 for ICP; *r* = −0.30, *P* < 0.01 for spouse) (4) Invalidation by spouse was negatively correlated with marital satisfaction in spouses (*r* = −0.28, *P* < 0.01) and negatively correlated with perceived spousal support reported by the ICP (*r* = −0.27, *P* < 0.01)	4
Cano, Leong, Williams, May, and Lutz	2012	Observational	95 mixed-sex couples	ICPs: 51.67 y (12.47) Partners: 51.62 y (13.07)	ICPs: 52.6% female Partners: 47.4% female	ICPs: 46.3% Caucasian, 46.3% African American, 2.1% American Indian/Alaskan Native, 5.3% mixed, 9.5% Hispanic/Latino Partners: 50.5% Caucasian, 46.3% African American, 2.1% Asian, 1.1% mixed, 9.5% Hispanic/Latino	Couples in which 1 partner reported chronic pain: disc or spine problems (55.8%), osteoarthritis (38.9%), other muscle or joint conditions (27.4%), fibromyalgia (7.4%), bone fractures (5.3%)	Validation (=behaviors and verbalizations that convey acceptance and attempted understanding) vs invalidation (=statements or behaviors that convey contempt, disrespect, and nonacceptance)	2 × 10 min videotaped marital interaction about the impact of pain on their lives Raters identified ICPs' disclosures of pain-related distress and coded spouses' responses as validation, invalidation or neutral using a manual developed for this study	(1) Pain-related measures: Pain Catastrophizing Scale (PCS), Pain Behavior Checklist (PBCL) (2) Depressive and anxiety symptoms: Mood And Anxiety Symptom Questionnaire (MASQ) (3) Relationship distress: DAS, survey of pain Attitudes—solicitous subscale (4) Pain-specific spousal support: MPI	(1) Invalidation was positively associated with ICP and spouse helplessness catastrophizing (*r* = 0.32, *P* < 0.01 and *r* = 0.28, *P* < 0.03, respectively), affective distress pain behaviors as reported by ICPs (*r* = 0.25; *P* < 0.05), and spousal anxiety (*r* = 0.27, *P* < 0.03) (2) Validation: no significant correlations with any of the variables	4
Gauthier, Thibault, and Sullivan	2008	Observational	58 couples	Total sample: 40 y (range 20–58)	ICPs: 28 females; 30 males	Not specified	Couples in which 1 partner deals with chronic neck or back pain	Pain-related empathic accuracy (=accurately inferring the ICP's level of pain)	ICPs were videotaped while performing a lifting task (=several trials of lifting canisters filled with sand). ICPs indicated their level of pain (0–10) for each trial. Spouses later viewed the videos and inferred the pain of their partners (1) Discrepancy index: overall difference between ICPs' self-reported pain levels and their spouses' estimations (2) Covariation index: within-couples correlations between the ICP's reported pain levels and the spouse's estimations across trials	(1) Pain-related measures: MPI/MPI-S, PCS, McGill Pain Questionnaire (MPQ), Pain Disability Index (PDI), Tampa Scale of Kinesiophobia (TSK) (2) Depression: beck Depression inventory-II (BDI-II) (3) Relationship adjustment: DAS	(1) The discrepancy index correlated positively with MPQ (*r* = 0.31, *P* < 0.05), PCS (*r* = 0.27, *P* < 0.05), TSK (*r* = 0.38, *P* < 0.01), and PDI (*r* = 0.41, *P* < 0.01). For these pain-related questionnaires, no significant correlations emerged with the discrepancy index (2) Regarding the MPI, the covariation index was associated with higher levels of interference of pain (*r* = 0.32, *P* < 0.05), higher levels of affective distress (*r* = 0.35, *P* < 0.05), lower levels of social activity for the partner with pain (*r* = −0.34, *P* < 0.05), and reduced perception that his or her spouse engaged in distracting responses (*r* = −0.27, *P* < 0.05). No significant correlations emerged with the discrepancy index (3) Regarding the MPI-S, the covariation index was associated with lower levels of social activity (*r* = −0.39, *P* < 0.05). The discrepancy index was positively associated with higher scores on the punitive subscale (*r* = 0.28, *P* < 0.05) (3) No significant correlations with pain-related empathic accuracy were found for relationship variables and both ICPs' and spouses' depression	4
Gauvin, Smith, Chamberlain and Pukall	2019	Observational	8 mixed-sex couples	ICPs: 25.25 y (4.59) Partners: 28.13 y (5.54)	ICPs: 100% female	ICPs: Caucasian (7); South Asian (1) Partners: Caucasian (6); South Asian (2)	Couples in which the female partner deals with provoked vestibulodynia (PVD, a recurrent vulvovaginal pain condition)	Empathic responses (=perspective-taking and positive physical touch that helps the partner feel understood and validated)	7-min videotaped discussion on an issue that the female partner had rated as moderately distressing Raters coded empathic responses using the acceptance code from the Rapid Marital Interaction Coding System (RMICS)	(1) Self-disclosure: RMICS self-disclosure code (2) Relationship satisfaction: DAS (3) Sexual satisfaction: Golombok-Rust Inventory of Sexual Satisfaction (GRISS)	(1) A greater number of one's own and one's partners empathic responses was associated with higher self-reported relationship satisfaction (β = 16.13, *P* < 0.05 for own empathy, β = 28.75, *P* < 0.05 for partner empathy) (2) Moderation effect: The effect of one's partners level of empathy on relationship satisfaction was stronger for men than women (β = 13.63, *P* < 0.05) (3) No effects of self-disclosure on relationship satisfaction (4) A greater number of one's own empathic responses was associated with better sexual satisfaction (β = −11.89, *P* < 0.05), while a greater number of one's own self-disclosures was associated with lower sexual satisfaction (β = 13.01, *P* < 0.01) (5) No significant effects of partner empathy or partners' self-disclosure on sexual satisfaction	4
Hemphill, Martire, Polenick, and Stephens	2016*	Longitudinal: Baseline (T1) 6 mo after BL (T2) 18 mo after BL (T3)	152 couples Data available for the 6 mo period (T1-T2): 139 couples Data available for the 12 mo period (T2-T3): 127 couples	ICP: 65.78 y (9.99) Partners: 65.32 y (12.02)	ICPs: 58.55% female Partners: 40.79% female	ICPs: 86.84% White, 11.18% Black Partners: 84.87% White, 11.84% Black	Couples in which 1 partner has knee osteoarthritis	Spouse responses to ICPs' pain: Empathic responses vs Nonempathic responses (solicitous, punishing)	Spouses self-reported the frequency of empathic responses on a scale by Stephens et al. (2006) and solicitous and punishing responses on subscales from the MPI	(1) Confidence in arthritis management: arthritis self-efficacy scale rated by ICPs and their spouses (ie, spouse confidence for patient's arthritis management) (2) Functional limitations: Western Ontario McMaster Universities Index—physical function subscale (3) Physical activity: Items from the Yale Physical Activity Survey (4) Pain severity: single-item 5-point scale	(1) Mediation effect of spouse confidence on change in patients' functional limitations and activity over 6 mo (T1-T2): Higher spouse confidence predicted increases in empathic responses (*B* = 0.01, *P* = 0.01), which were associated with reductions in patients' functional limitations (*B* = −2.21, *P* = 0.03) and increases in physical activity (*B* = 4.72, *P* = 0.01). No significant indirect effects were found through solicitous or punishing, responses (2) Mediation effect of spouse confidence on change over 12 mo (T2-T3): Higher spouse confidence predicted decreases in solicitous responses (*B* = −0.02, *P* = 0.01), which were related to decreases in patients' functional limitations (*B* = 2.36, *P* = 0.01) and increases in physical activity (*B* = −3.29, *P* = 0.05). No significant indirect effects were found through punishing or empathic responses	5
Leong, Cano, and Johansen	2011	Observational	78 mixed-sex couples	ICP: 53.78 y (12.09) Partners: 54.71 y (12.25)	ICPs: 58% female Partners: 42% male	ICPs: 53% Caucasian, 38% African American, 9% other Partners: 50% Caucasian, 42% African American, 8% other	Couples in which at least 1 partner reported chronic musculoskeletal pain: osteoarthritis (32.1%), disc problems (19.2%), fibromyalgia (5.1%), other nerve problems (5.1%), other musculoskeletal pain (3.8%), other (9%) In 48.7%, both partners were affected by chronic pain (ICP = the partner with the more severe/disabling pain)	Validation (=spouse responses that convey acceptance and an attempt to understand his or her partner's experiences) vs invalidation (=spouse responses that convey disrespect, contempt, or non-acceptance of the other person's experiences)	15-min marital discussion about a topic of disagreement (not necessarily related to pain) Coders used the Specific Affect Coding System (SPAFF) to assess frequency and sequences of validation and invalidation Frequency: number of validating/invalidating responses per speaker turn Sequences: Invalidation followed by validation, invalidation followed by neutral, invalidation followed by invalidation	(1) Depressive symptoms: MASQ—nonspecific depression subscale, Anhedonic Depression subscale (2) Pain: MPI—Pain Severity Subscale (3) Marital satisfaction: DAS	Sequences (1) Overall sample: Sequences were not significantly related to pain severity, depression, or marital satisfaction (2) Moderation effect of gender: In male patient couples, a stronger sequence of spouse invalidation followed by patient invalidation was significantly related to greater patient pain (*r* = 0.48, *P* < 0.01). A stronger sequence of spouse invalidation followed by patient neutral was related to lower patient pain (*r* = −0.35, *P* < 0.05) and lower spouse depression (*r* = −0.42, *P* < 0.05). A stronger sequence of patient invalidation followed by spouse validation was associated with lower pain rated by the spouse (*r* = −0.34, *P* < 0.05). No such relationships were found for female patient couples Frequency (3) Overall sample: For ICPs and spouses, higher base rates of invalidation were significantly associated with lower marital satisfaction (ICPs: *r* = −0.33, *P* < 0.01; spouses: *r* = −0.22, *P* < 0.05). Invalidation was unrelated to pain and depression. Validation was unrelated to pain, depression, and marital satisfaction (4) Moderation effect of gender: In male patient couples, higher rates of spouse validation were associated with greater patient pain (*r* = 0.44, *P* < 0.01), but lower marital satisfaction (*r* = −0.35, *P* < 0.05) (5) Moderation effect of gender: In female patient couples, higher rates of invalidation by both partners were significantly related to higher spouse depression (*r* = 0.41, *P* < 0.01 for patient invalidation; *r* = 0.32, *P* < 0.05 for spouse invalidation)	6
Martire, Stephens, Mogle, Schulz, Brach, and Keefe	2013*	Longitudinal: End-of-day diary assessments and accelerometer for 22 days	141 couples	ICPs: 65.4 y (9.8) Partners: 65.1 y (11.5)	ICPs: 43% male Partners: 58% male	ICPs: 87% Caucasian Partners: 86% Caucasian	Couples in which 1 partner has knee osteoarthritis	Activity-related autonomy support (=actions characterized by empathy and understanding for an individual's situation and the provision of choices for making health behavior changes)	At the end of each day, ICPs reported the extent to which their spouses “showed understanding for how physically active I wanted to be,” “respected my decisions or choices about physical activity”, and “listened to how I would like to do things with regard to being more or less physically active” (3 items)	(1) Physical activity: number of minutes in moderate-intensity activity and number of steps taken (2) Spouse influence: activity-related Persuasion and Activity-related pressure measured with 4 and 2 items, respectively (3) Pain: Rapid Assessment of Disease Activity In Rheumatology (4) Marital tension and enjoyment: Single items	(1) On days when ICPS reported more autonomy support from their spouses, ICPs engaged in significantly more moderate-intensity physical activity (estimate = 5.19, *P* < 0.05) and took more steps (estimate = 372.87, *P* < 0.01) (2) Spouses' daily persuasion and pressure were not associated with ICPs' physical activity or steps, except for male patient couples (moderation effect of gender): Male ICPs were less active on days when they reported more pressure from their spouses (estimate = −20.5, *P* < 0.01)	4
Martire, Zhaoyang, Marini, Nah, and Darnall	2019*	Longitudinal: baseline interviews and 22-day diary assessments (3 times per day)	144 couples	ICPs: 65.5 y (9.59) Partners: 65.3 y (11.44)	ICPs: 42.4% male Partners: 58.3% male	ICPs: 86.8% White Partners: 85.4% White	Couples in which 1 partner has knee osteoarthritis	Empathic responses (= showing affection, understanding patients' feelings about the pain, providing attention) vs nonempathic responses (punishing, solicitous)	Empathic responses: 3 items adapted from Stephens et al. (2006) Solicitous and punishing spousal responses: 3 items each, adapted from the MPI Responses were rated by the ICP at the end of each day	(1) Pain catastrophizing: 2 items from the Coping Strategies Questionnaire rated by the ICP in the morning (2) Negative affect: 5 items rated by ICPs & spouses 3 times per day (3) Depressive symptoms: Center for Epidemiologic studies depression scale—short form (4) Pain: Rapid Assessment of Disease Activity In Rheumatology	(1) Patients' morning pain catastrophizing was related to greater spousal negative affect throughout the day (estimate = 0.026, *P* = 0.045), but not spousal responses (empathic, solicitous, or punishing) (2) Punishing spouse responses predicted greater patient catastrophizing the next morning (estimate = 0.190, *P* = 0.031). Empathic and solicitous responses did not significantly predict next-day catastrophizing	4
Rosen, Bois, Mayrand, Vannier, and Bergeron	2016	Observational	50 couples (49 mixed-sex, 1 same-sex)	ICPs: 24.5 y (4.03) Partners: 26.1 y (5.7)	ICPs: 100% female	Not specified	Couples in which the female partner deals with vulvodynia	Empathic responses (=verbal attention, attempt to understand the other, acceptance) vs nonempathic responses (eg, criticizing the speaker or ignoring the speaker's expression of distress)	A filmed marital conversation about vulvodynia (1) Perception of spouse's empathic responses: 3 items about feeling understood, accepted and cared for; rated by the participants after the discussion (2) Observed empathic responses: rated by an observer using the Empathic Response Card-Sort (ERCS)	(1) Perceived disclosure: 8 items about the perception of one's own and the partner's disclose, respectively; rated after the discussion (2) Observed disclosure: rated by an observer using the Disclosure Coding System (3) Intercourse pain intensity: NRS (4) Quality of Life: Skindex-29—adapted version (5) Relationship adjustment: DAS—brief version	(1) Perceived empathy of ICPs was associated with higher ICPs' quality of life (*r* = 0.36, *P* < 0.05) and higher relationship adjustment reported by both the ICP (*r* = 0.35, *P* < 0.05) and their partner (*r* = 0.33; *P* < 0.05) (2) Perceived empathy of the partner was associated with higher ICPs' quality of life (*r* = 0.33, *P* < 0.05) and higher relationship adjustment reported by both the ICP (*r* = 0.52, *P* < 0.001) and their partner (*r* = 0.40, *P* < 0.01) (3) Observed empathy of ICPs was associated with higher relationship adjustment reported by both the ICP (*r* = 0.51, *P* < 0.001) and their partner (*r* = 0.54, *P* < 0.001) (4) Observed empathy of the partner was associated with higher ICPs' quality of life (*r* = 0.36, *P* < 0.05) and higher relationship adjustment reported by both the ICP (*r* = 0.29, *P* < 0.05) and their partner (*r* = 0.38, *P* < 0.01) (5) None of the empathy measures correlated with pain	5
Stephenson, DeLongis, Esdaile, and Lehman	2014	Longitudinal: Baseline, 1 y later	133 couples	ICPs: 61.9 y (12.7) Partners: *M* (*SD*) not specified; 81.8% of the partners were older than 50 y	ICPs: 72.9% female	ICPs: 94.5% White Partners: 95.5% White	Couples in which 1 partner deals with rheumatoid arthritis	Empathic responses (1) Cognitive/affective strategies: perspective taking, vicarious experiencing of another's concerns (2) Behavioral strategies: listening, providing comfort or support	ICPs rated their spouses' empathic responding on a 10-item scale by O'Brien and DeLongis (1996)Spouses rated their own empathic responding on the same scale Both measures were taken at baseline	(1) Functional limitations: The Disabilities of the Arm, Shoulder, and Hand (DASH) questionnaire (2) Marital quality: Quality Marriage Index (QMI) (3) Depression: Center for Epidemiologic Studies Depression Scale (CES-D)	(1) Main effects on ICP disability at follow-up: Greater spouse depressive symptoms (β = 0.16, *P* = 0.004), but not ICP depressive symptoms nor spouse empathic responding predicted greater ICP disability after 1 y (2) Moderation effect: ICPs' perception of their spouse's empathic responding moderated the effect of spouse depression on ICP disability (β = −0.11, *P* = 0.045): A higher perception of spousal empathic responding reduced the effect of spouse depression on ICP disability (3) Main effects on ICP marital quality at follow-up: Lower marital quality at follow-up was predicted by greater ICP (β = −0.12, *P* = 0.044) and spouse depressive symptoms (β = −0.14, *P* = 0.018). ICPs' perception of spouse empathy did not predict later marital quality (4) Again, a higher perception of spousal empathy reduced the effects of both ICP depressive symptoms (β = 0.12, *P* = 0.040) and spouse depressive symptoms (β = 0.11, *P* = 0.049) on marital quality at follow-up (moderation effect)	4
Wilson, Martire, and Sliwinski	2017*	Longi-tudinal: Baseline (T1), followed by a 22-days daily diary assessment 6 mo after BL (T2) 18 mo after BL (T3)	145 couples	ICPs: 65.6 y (9.8) Partners: 65.3 y (11.5)	ICPs: 43% male Partners: 58% male	ICPs: 88% White Partners: 86% White	Couples in which 1 partner has knee OA	Empathic vs nonempathic responsivity: Empathic (= showing affection, understanding, providing attention) vs nonempathic (punishing, solicitous) reactions to changes in the ICPs' verbal pain expression	Empathic responses: 3 items adapted from Stephens et al. (2006) Solicitous and punishing spousal responses: 3 items each, adapted from the MPI Responses were rated by the ICP at the end of each day	(1) ICP verbal pain expression: assessed by the spouse with a single item at the end of each day (2) ICP physical function: Short physical Performance Battery at T1, T2, and T3 (3) ICP pain severity: assessed by the ICP with a single item at the end of each day	(1) Patients in dyads that exhibited higher empathic responsivity showed steeper linear improvements in physical function across 18 mo (estimate = 0.337, 95% CI = 0.039–0.635, *P* = 0.027) (2) Solicitous nor punishing responsivity were unrelated to change in physical function	4

* Data in these studies are from the same sample.

BL, baseline; ICP, individual with chronic pain; QA, quality assessment.

In the pre-registered protocol, it was planned to divide the included studies between 2 researchers for independent extraction. However, to improve the reliability of the extraction process, the process was modified as follows: One reviewer (H.G.) conducted data extraction for all studies. In addition, a second reviewer (K.T.) randomly selected 50% of the studies for independent data extraction and risk-of-bias assessment. H.G. and K.T. compared their results and resolved discrepancies through discussion. For the remaining half of the studies, K.T. double-checked the initial extraction. This was the only deviation from the pre-registration. The data from the included studies were synthesized narratively. The size of correlation coefficients is evaluated according to Cohen^13^: small (r ≈ 0.1), medium (r ≈ 0.3), and large (r ≈ 0.5).

### 2.4. Risk-of-bias assessment

The Risk of Bias Utilized for Surveys Tool (ROBUST)^44^ was applied to assess the methodological quality of the included studies. The ROBUST tool evaluates 8 key domains of bias using dichotomous criteria, such as adequacy of sample size or transparency in data management. Each criterion can either be met, receiving a score of 1 (indicating a low risk of bias), or not met, receiving a score of 0 (indicating a high risk of bias). A final score for each study is calculated, ranging from 0 to 8, with higher scores indicating a lower risk of bias. H.G. and K.T. resolved ambiguities in risk-of-bias assessment through consensus.

## 3. Results

A total of 434 published articles were initially identified from the database searches with the English search terms. No results could be found in any of the databases using the German search terms. After removing 110 duplicate articles, 324 studies underwent title and abstract screening, and 36 underwent full-text review. During the full-text screening, 26 studies were excluded for different reasons listed in the flowchart (Fig. 1). A list of the respective studies and a detailed description of the specific reason for their exclusion is provided in the supplementary material, http://links.lww.com/PR9/A349. Three additional studies were identified through citation searching; however, one was excluded because it was a conference proceeding, and another study was excluded for measuring empathy as an outcome variable, which is inconsistent with our PICO model (see Supplements, http://links.lww.com/PR9/A349). Finally, a total of 11 articles were included in our systematic review.

### 3.1. Study characteristics

Included studies were conducted between 2008 and 2019. Five studies used observational designs, where interactions between partners were observed and systematically coded. One study used a purely correlational design. Another 5 studies used longitudinal designs, using either daily diary methods or assessments taken at multiple distinct time points. Notably, 4 of these 5 studies reported different data from the same sample of patients with knee osteoarthritis and their spouses. To facilitate the tracking of this information, references to the 4 studies from the same sample are marked with an asterisk (*) in the following.

The sample sizes of the included studies varied considerably, ranging from 8 couples to 145 couples. Demographic data reported across studies also show inconsistencies. Aligning with epidemiological studies on the prevalence of chronic pain,^48^ most studies (n = 8, although 4 analyzed the same sample) included middle-aged adults with the mean ages between 50 and 65 years. The remaining 3 studies investigated younger samples. Regarding ethnic diversity, 4 studies included African American, Caucasian, and other racial backgrounds. However, several studies did not report ethnicity (n = 2 studies) or only specified percentages for White and Black participants (n = 1 study). Four of the studies mentioned only the percentage of White participants.

Regarding health status, the most common diagnosis for the ICPs in the included studies was knee osteoarthritis (n = 4 studies, but on the same sample). Apart from that, several studies included patients with various forms of chronic musculoskeletal pain. One study included patients who had chronic musculoskeletal pain with unspecified details, while 3 studies described more specific musculoskeletal pain conditions, such as low back pain, neck, and shoulder pain. Two studies examined pain conditions that specifically affected female patients: one study focused on provoked vestibulodynia and another on vulvodynia. One study included participants with rheumatoid arthritis. Table 1 summarizes an overview of all data extracted from the included studies.

### 3.2. Study quality

Of the 11 studies included, 10 received a score of 4/8 or 5/8 on the ROBUST scale, reflecting a uniformly moderate risk of bias. With a score of 6, the lowest risk of bias was found in the study by Leong et al.^34^ Please refer to the Supplementary material, http://links.lww.com/PR9/A349 to review our risk-of-bias scoring for each study.

### 3.3. Conceptualization and measurement of empathy

Empathy was conceptualized and measured in various ways across the included studies. The most common conceptualization was defining empathic behavior from the spouse as validation. In 3 studies, validation was considered a response, which included acceptance of the partner and an attempted understanding of their thoughts, feelings, and experiences.^7,11,34^ Couples were invited to the laboratory and instructed to discuss a specific topic. In 2 studies,^7,11^ the interactions focused on the partner's pain experience and its impact on their lives, while 1 study^34^ instructed participants to discuss a topic of disagreement. No specific instructions were given regarding empathy or validation. Spousal interactions were videotaped and systematically coded by trained observers. Although all 3 studies aimed to identify validating and invalidating behaviors, each used different coding systems (Table 1).

Two other studies also assessed empathic responses by observing and coding in-lab spousal interactions, although the theoretical framework differed: Gauvin et al.^23^ defined empathy as acceptance behaviors, such as perspective-taking and positive physical touch, and used the Rapid Marital Interaction Coding System,^27^ for coding verbal and nonverbal behaviors from the couples' interactions. Rosen et al.^53^ understood empathy as a response involving understanding, validation, and caring. They took a dual approach by developing and incorporating both observational and self-reported measures of empathy. Specifically, an observer rated the couples‘ videotaped interaction using the Empathic Response Card-Sort, while participants answered 3 items referring to the degree to which they felt understood, accepted, and cared for by their partner during the discussion.

Hemphill et al.^26^* asked patients’ spouses to indicate the extent of empathic spousal responses on a scale from Stephens et al.^59^ The original 7 items assess supportive partner communication and empathic understanding regarding the partner's pain in the past month, eg, “tried to put yourself in [the patient's] situation,” “showed [the patient] affection to comfort him/her.” In their daily diary survey, Martire et al.^40^* and Wilson et al.^65^* used 3 items from this scale to assess empathy from the patients' and/or spouses' perspective at the end of each day.

A solely cognitive approach was taken by Gauthier et al.,^22^ who measured pain-related empathic accuracy, ie, the ability of the spouse to accurately infer the partner's internal pain states from videotaped sequences of the patient performing a lifting task. Accuracy was operationalized through a Discrepancy Index, which measured the difference between self-reported pain and the spouse's estimation, and a Covariation Index, which assessed the spouse's sensitivity to fluctuations in the patient's pain over different trials of the lifting task.

Martire et al.^39^* took a different approach by conceptualizing empathy through autonomy support, defined as actions demonstrating empathy and understanding for the partner's situation and providing choices for health behavior changes. This aspect of empathy was measured by 3 items assessing the extent to which the spouse (1) showed understanding for how physically active the partner wanted to be, (2) respected the partner's decisions about physical activity, and (3) listened to the partner's preferences regarding physical activity.

Finally, Stephenson et al.^60^ used a 10-item self-report scale by O'Brien and DeLongis^45^ to measure empathic responding by capturing cognitive-affective aspects (eg, “Imagined myself in the other person's shoes”) and behavioral strategies (eg, “Tried to comfort the other person by showing my positive feelings for him/her”).

### 3.4. Association between empathy and pain-related outcomes

Four studies demonstrated a positive effect of empathy on pain-related outcomes, specifically physical functioning. For example, during 22 days of daily diary assessment, activity-related autonomy support was associated with more moderate-intensity physical activity and an increased step count for patients. By contrast, persuasion and pressure from the spouse did not relate to the level of physical activity in the patients.^39^* Male ICPs were even less active on days when they reported more activity-related pressure from their spouses. Longitudinally, Wilson et al.^65^* reported that ICPs, whose spouses exhibited greater empathic responsivity to changes in the ICPs' verbal expression of pain at baseline, showed steeper improvements in their physical function over the following 18 months. Similarly, Hemphill et al.^26^* longitudinally examined the mediating role of spouse responses to pain. Specifically, spouse confidence in patients' disease management at baseline was indirectly linked to improvements in patients' functional limitations and activity levels after 6 months, mediated through greater empathic responses to the patients' pain. This effect, however, was not observed over a twelve-month follow-up period: Although higher spousal confidence was still related to better patient functioning and physical activity, this effect was mediated through a decrease in solicitous responses, potentially implicating a gain in independence in those patients whose spouses trusted the disease management abilities of their partners. These 3 studies^26^*^,^^39^*^,^^65^* must be interpreted in light of the fact that they all analyzed data from the same sample. In a longitudinal design with a different sample, Stephenson et al.^60^ observed that spouses' empathic responding at baseline reduced the impact of spousal depressive symptoms on patients' disability 1 year later.

However, 3 studies showed nonsignificant or even disadvantageous associations of empathy with pain-related outcomes: Martire et al.^40^* examined the association between ICPs' pain catastrophizing in the morning and spouses' responses throughout the day over a 22-day diary assessment period. While ICPs' pain catastrophizing in the morning and spouses' negative affect and punishing responses (ie, ignoring the patient, acting frustrated, or seeming irritated) throughout the day were related, there was no evidence that spouses' empathic or solicitous responses preceded or followed patients' catastrophizing. Similarly, Leong et al.^34^ found no significant cross-sectional association between observed validation and self-reported pain severity in their study. However, a moderation effect of sex emerged: Higher rates of spouse validation correlated moderately with greater pain, specifically in male patient couples. Gauthier et al.^22^ observed small-to-medium–sized negative correlations of higher pain-related empathic accuracy with different pain- and health-related variables. That is, the more accurately spouses inferred their partners' pain, the higher the degree of interference of pain, affective distress, and reduction of social activities in the patients. As a possible explanation, the authors propose that empathic accuracy could sometimes lead to adverse outcomes by overwhelming the spouse's emotional resources and impairing their ability to provide support. However, other explanations are possible (see discussion). In sum, most studies confirmed an association between spouse empathy or validation and ICPs' pain and functioning. However, the evidence remains inconclusive due to null results and contradictory findings, particularly from the study with the lowest risk of bias.^34^

### 3.5. Empathy and relationship outcomes

Similarly to pain-related outcomes, several studies showed a positive association between empathy and relationship outcomes. Cross-sectionally, observed validating and empathic responses were positively correlated with self-reported marital satisfaction in the studies by Cano et al.^7^ and Gauvin et al.,^23^ who also found that the effect of one's partner's level of empathy on relationship satisfaction was moderated by sex, being stronger for men than women. Similarly, Rosen et al.^53^ reported that perceived and observed empathic responses correlated with higher relationship adjustment reported by ICPs and their partners to a medium extent. Regarding empathy's moderating role, the study by Stephenson et al.^60^ demonstrated that higher perceived empathic responding at baseline attenuated the effects of ICPs' and spouses' depressive symptoms on marital quality at a one-year follow-up.

In contrast to expectations, a study that reported a link between spouse validation with greater patient pain (see above) also revealed a negative small-to-medium effect of frequent spouse validation on marital satisfaction. However, both effects were observed in male patient couples only.^34^ Similarly, Cano et al.^11^ found no significant correlation between observed validation and relationship outcomes. Gauthier et al.^22^ also did not report any significant associations between pain-related empathic accuracy and relational outcomes, such as dyadic adjustment and length of the relationship.

Only 1 study specifically assessed sexual satisfaction within the domain of relationship satisfaction. In this study, which included female patients with provoked vestibulodynia, a recurrent vulvovaginal pain condition, a greater number of observed empathic responses was associated with higher sexual satisfaction, whereas greater observed self-disclosures were associated with lower sexual satisfaction. No effects of partner empathy or partners' self-disclosure on sexual satisfaction were found.^23^

In sum, most studies linked spouse empathy or validation to marital functioning, although some ambiguity remains.

### 3.6. Invalidation

Eight studies also examined the effects of invalidating or nonempathic interactions on various outcomes for patients and spouses. Invalidating reactions could include, for instance, inattentiveness, telling the spouse what they should think or feel, or putting them down.^7^ To a small-to-medium extent, observed invalidation by both partners was associated with lower marital satisfaction^7,34^ and reduced perceived spousal support.^7^ In female patient couples, higher rates of invalidation by both partners were also significantly moderately related to higher spouse depression.^34^ In addition, Stephenson et al.^60^ identified a moderating role of empathy in the longitudinal association between depression and marital quality: patient and spouse depression at baseline were linked to poorer marital quality at follow-up when spouses' empathic responding was low. The same study also reported that spouse depression was linked to greater patient functional impairment 1 year later in cases when the spouse showed low empathic responding. The results of another repeated-measurement study demonstrated that punishing spouse responses on 1 day predicted greater patient catastrophizing the next morning.^40^* Cross-sectionally, Cano et al.^11^ also observed that invalidation was moderately associated with helplessness catastrophizing in both partners, as well as with increased distress-related pain behaviors reported by the ICP and heightened anxiety in the spouse.

On the other hand, Hemphill et al.^26^* observed no adverse effect of punishing responses (eg, getting angry with or ignoring the partner) on the trajectory of the patient's functional limitations over 6 and 12 months. Analyzing the same sample, Wilson et al.^65^* also found no significant effect of solicitous or punishing responsiveness, ie, the degree to which spouses' solicitous or punishing reactions calibrate to changes in the ICPs' pain expression, on changes in ICPs' physical function over time.

## 4. Discussion

This systematic review reveals the important role of empathy in couples dealing with chronic pain. Several reviewed studies demonstrated that empathic or validating interactions positively affected pain-related outcomes, such as improved physical functionality and activity^26^*^,^^39^*^,^^60,65^* as well as relationship outcomes, including improved relationship satisfaction and quality.^7,23,53,60^ These results were evident for self-reported and observed measures of empathy and validation and could be found in cross-sectional and longitudinal study designs. The findings align with theoretical frameworks such as the interpersonal process model of intimacy,^52^ which posits that empathic responses deepen relational bonds and foster greater emotional intimacy. Contrary to the predictions of the operant model of pain,^21^ empathic responses do not reinforce pain behavior and instead relate to adaptive patient outcomes.^18^ In particular, we found no evidence of a disadvantageous effect of solicitousness on chronic pain or functioning.

However, not all findings supported the positive effects of empathy. In male patient couples, Leong et al.^34^ found that higher levels of spouse validation were related to greater pain and lower marital satisfaction. Another negative effect was reported by Gauvin et al.,^23^ who found an association between self-disclosures and sexual dissatisfaction. The authors suggest several explanations for their findings, such as a reduction of sexual desire and pleasure due to self-disclosures about negative emotions. Individuals who frequently self-disclose might also report their sexual dissatisfaction more openly.^23^ Surprisingly, this study was the only one explicitly addressing sexual functioning, although sexual difficulty among patients with chronic pain (also beyond genital pain) is common and burdensome.^20^ While reasons such as exacerbating pain, physical limitations, or medication are discussed in the literature,^1^ future studies should shed more light on socio-affective factors that influence sexual satisfaction in chronic pain couples.

The study by Gauthier et al.^22^ contradicted the expectation that empathic understanding would lead to more adaptive outcomes. According to their findings, higher pain-related empathic accuracy was linked to greater interference of pain in the patient's life, increased affective distress, and reduced social activity. The authors propose an indirect association between higher empathic accuracy and patients' dysfunction driven by empathic distress of the spouses and, consequently, less spousal support.^22^ Alternatively, patients with more severe pain conditions could express their pain and disability more strongly and thereby facilitate estimation, resulting in increased accuracy for patients with more pronounced symptoms. However, other findings suggest that the association between accuracy and pain severity reverses when the patient's pain persists over time,^33^ indicating that, in the long term, spouses of ICPs with high pain severity may become less empathically accurate to reduce their emotional burden. In addition, empathic accuracy alone does not guarantee beneficial outcomes: In healthy couples, higher accuracy was linked to declines in subjective closeness when they expressed relationship-threatening thoughts or feelings during an in-lab discussion.^56^

Although 2 studies found no significant effects, 6 studies consistently showed the detrimental effects of invalidating or nonempathic responses on marital or relationship quality,^7,34,60^ along with various affective and cognitive aspects^11,34,40^* and physical functioning.^60^ Similarly, previous research has shown that a lack of empathy or validation in social relationships is associated with a wide range of negative outcomes, such as reduced mental well-being,^12^ poorer physical health,^30^ and reduced social functioning.^24^ These negative effects may be explained by an impaired ability to regulate emotions when an individual encounters an invalidating environment.^4^ Invalidating communication could also signal a lack of social safety,^24^ whereas feeling supported, accepted, and connected is essential for maintaining health and well-being.^58^

### 4.1. Limitations

This systematic review has several limitations that should be considered when interpreting its findings. First, the studies included vary in their measurement and conceptualization of empathy in couples coping with chronic pain. While some share overlapping components, such as validation, they also differ in key aspects. Some studies relied on self-reported measures, while others used observational coding systems to assess empathic behaviors. These diverse methodologies make it difficult to compare results across studies and impede meta-analytical calculations, which would have added value to this narrative summary. Second, the samples lacked diversity in age, sexual orientation, and ethnicity, with most participants being middle-aged, heterosexual Caucasian couples. Four studies even reported different data from the same study cohort, leading to an overrepresentation of older patients with knee osteoarthritis and their spouses in this review. This lack of sample diversity restricts the applicability of the findings to broader populations, including younger couples or those from different ethnic backgrounds. Furthermore, the participants in the included studies represent a small scope of chronic pain conditions, with musculoskeletal disorders and vulvovaginal pain being particularly prevalent. Future research should include a wider variety of chronic pain conditions, eg, neuropathic pain or fibromyalgia, to explore whether empathy has similar effects across different pain syndromes.

The review process itself also has limitations. The literature search did not include grey literature, and only 1 reviewer conducted the screening process in an unblinded manner. This could have introduced selection bias or reduced the reliability of study selection. Future reviews may also benefit from using a broader range of search terms to capture different facets of empathy, potentially enabling comparisons between affective and cognitive approaches.

### 4.2. Implications

Given the importance of social influences on chronic pain, interventions might benefit from involving relatives in the therapeutic process. Moreover, specific treatments for couples, including empathic responding exercises, are available.^8^ Martire et al.^38^ indeed demonstrated that couple-based interventions provide greater benefits for managing chronic pain than individual approaches alone. Based on insights from this systematic review, specifically incorporating empathy and validation training into interventions for couples coping with chronic pain could prove effective. For example, Edlund et al.^17^ found that a brief training session in validation increased validating behaviors in couples coping with chronic pain, as well as in sibling and parent–child pairs. In the context of children's pain, validation is even discussed as a mechanism for preventing the development of chronic pain.^63^

One study reported negative outcomes of spousal validation specifically for male patient couples,^34^ and another study found that one's partner's level of empathy affected relationship satisfaction more strongly for men than women.^23^ In addition, social support seems to affect men and women differently.^64^ Thus, tailoring interventions to account for gender differences seems necessary. Finally, addressing invalidating behavior within relationships of chronic pain couples is essential. Interventions should aim to reduce these behaviors actively, as studies in healthy individuals demonstrated that invalidating feedback predicts increased threat-related emotional and physiological arousal and reduced social engagement behaviors.^24^ Avoiding invalidation is also crucial for healthcare professionals, who interact with ICPs, to maximize patient satisfaction and foster good communication.^36,62^

Future studies should work to refine and standardize the ways empathic or validating responses are measured in couples coping with chronic pain. Specifically, adding subjective and objective measurement tools would allow exploring whether the mere perception of empathy is sufficient to produce positive outcomes or if objectively empathic responses are necessary. Relatedly, some researchers suggest that the pain patient's perception of their partner's support and responsiveness may be more important than their actual behavior.^37,52^ Only 2 studies in this review^53,60^ examined spouses' perception of empathic responding and found positive effects. Included studies also mainly focused on the spouse's empathic responses toward the pain patient. Nevertheless, understanding others in a relationship is a mutual process, indicating that a comprehensive view of empathy in relationships also needs to account for reciprocity. Future research should expand on this.

It may also be valuable to differentiate between state and trait empathy in the context of chronic pain. Although the spouse of a partner dealing with chronic pain might generally be an empathic individual, they may still react less empathically to their partner's pain-related expressions in certain situations. As empathy and compassion are cognitively costly, people might avoid these responses in situations in which they perceive empathy as effortful and aversive.^5,54^ Considering empathy as a motivated decision^6^ provides an interesting perspective on the reported associations of empathy and pain or relationship satisfaction, respectively: Partners could more likely choose to respond in an empathic or validating manner if marital functioning is high and/or their partners are less limited by their pain. The correlational design of the included studies, however, precludes conclusions on the direction of associations.

Most of the included studies in this review captured a momentary state of empathy. Future research could offer valuable insights by comparing momentary assessments of empathy^49^ with spouses' trait levels of empathy, thereby enhancing our understanding of potential deviations and their underlying causes.

Another key area for future investigation is the need for more longitudinal studies to better understand how empathic behaviors evolve over time and their lasting impact on relationships and health outcomes. In addition, such studies could examine phenomena such as empathic distress or caregiver burnout^28,66^ and include various social interaction partners, eg, friends or family members, such as parents, children, or siblings.^25^

## 5. Conclusion

The findings from this systematic review suggest that empathy and validation generally have a positive impact on patients´ physical function, physical activity, relationship satisfaction, and relationship quality in couples with chronic pain. Conversely, the review also revealed that nonempathic and invalidating spouse behavior has largely detrimental effects on relationship quality, emotional well-being, and physical functioning of patients. Thus, incorporating empathy and validation training in interventions may be a promising tool in couples with chronic pain. Based on the literature presented in this study, we believe that the positive effects of empathy and the disadvantages of invalidation in a chronic pain context might not be restricted to intimate relationships. Anyone dealing with a person in pain, whether professionally or privately, should bear in mind that their experience is real and legitimate and that nobody should be invalidated for disclosing their (pain-related) thoughts or feelings. Clinicians who counsel spouses or family members of ICPs should recommend more empathic and validating responses to pain-related emotional disclosures and ensure that they consistently display these behaviors themselves. This could advance our understanding and treatment of chronic pain, and many patients and their loved ones will benefit.

## Disclosures

K.T., H.G., L.M.B., G.K., P.K., H.L.R., and A.M.F.R. have no conflicts of interest.

## Supplemental digital content

Supplemental digital content associated with this article can be found online at http://links.lww.com/PR9/A349.

## References

[1] Barr A Moore K Flegge LG Atsaphanthong E Kirby KE Craner JR. Predictors of sexual satisfaction among patients with chronic pain. Front Pain Res (Lausanne) 2024;5:1375546. 38638533 10.3389/fpain.2024.1375546 PMC11024270

[2] Bendahhou K Serhier Z Diouny S Simou M Mouzoun FZ Niyonsaba A Chemaou A Bennani Othmani M. Parental response to children's chronic pain. Cureus 2023;15:e39149. 37332473 10.7759/cureus.39149 PMC10275628

[3] Bodenmann G. Dyadic coping and its significance for marital functioning. In: Revenson TA Kayser K Bodenmann G, eds. Couples coping with stress: Emerging perspectives on dyadic coping. Washington, DC: American Psychological Association; 2005:33–49.

[4] Brandao T Brites R Hipólito J Nunes O. Perceived emotional invalidation, emotion regulation, depression, and attachment in adults: a moderated-mediation analysis. Curr Psychol 2023;42:15773–81.

[5] Cameron CD Hutcherson CA Ferguson AM Scheffer JA Hadjiandreou E Inzlicht M. Empathy is hard work: people choose to avoid empathy because of its cognitive costs. J Exp Psychol Gen 2019;148:962–76. 30998038 10.1037/xge0000595

[6] Cameron CD Rapier K. Compassion is a motivated choice. In: Sinnott-Armstrong W Miller CB, eds. Moral psychology: virtue and character. Cambridge: MIT Press; 2017:373–408.

[7] Cano A Barterian JA Heller JB. Empathic and nonempathic interaction in chronic pain couples. Clin J Pain 2008;24:678–84. 18806532 10.1097/AJP.0b013e31816753d8 PMC2562912

[8] Cano A Corley AM Clark SM Martinez SC. A couple-based psychological treatment for chronic pain and relationship distress. Cogn Behav Pract 2018;25:119–34. 29497271 10.1016/j.cbpra.2017.02.003 PMC5826564

[9] Cano A de C Williams AC. Social interaction in pain: reinforcing pain behaviors or building intimacy? PAIN 2010;149:9–11. 19892466 10.1016/j.pain.2009.10.010 PMC2834842

[10] Cano A Goubert L. What's in a name? The case of emotional disclosure of pain-related distress. J Pain 2017;18:881–8. 28163234 10.1016/j.jpain.2017.01.008 PMC5537014

[11] Cano A Leong LEM Williams AM May DKK Lutz JR. Correlates and consequences of the disclosure of pain-related distress to one's spouse. PAIN 2012;153:2441–7. 23059054 10.1016/j.pain.2012.08.015 PMC3494762

[12] Coady A Godard R Holtzman S. Understanding the link between pain invalidation and depressive symptoms: the role of shame and social support in people with chronic pain. J Health Psychol 2024;29:52–64. 37565664 10.1177/13591053231191919 PMC10757395

[13] Cohen J. Statistical power analysis for the behavioral sciences. Hillsdale, NJ: L. Erlbaum Associates, 1988.

[14] Craig KD. Social communication model of pain. PAIN 2015;156:1198–9. 26086113 10.1097/j.pain.0000000000000185

[15] Davis MH. Measuring individual differences in empathy: evidence for a multidimensional approach. J Personal Soc Psychol 1983;44:113–26.

[16] de Vignemont F Singer T. The empathic brain: how, when and why? Trends Cogn Sci 2006;10:435–41. 16949331 10.1016/j.tics.2006.08.008

[17] Edlund SM Carlsson ML Linton SJ Fruzzetti AE Tillfors M. I see you're in pain—the effects of partner validation on emotions in people with chronic pain. Scand J Pain 2015;6:16–21. 29911586 10.1016/j.sjpain.2014.07.003

[18] Edmond SN Keefe FJ. Validating pain communication: current state the science. PAIN 2015;156:215–9. 25599441 10.1097/01.j.pain.0000460301.18207.c2 PMC4477266

[19] Ferguson HJ De Lillo M Woodrow-Hill C Foley R Bradford EEF. Neural empathy mechanisms are shared for physical and social pain, and increase from adolescence to older adulthood. Soc Cogn Affect Neurosci 2024;19:nsae080. 39492751 10.1093/scan/nsae080 PMC11630255

[20] Flegge LG Barr A Craner JR. Sexual functioning among adults with chronic pain: prevalence and association with pain-related outcomes. Pain Med 2023;24:197–206. 35929084 10.1093/pm/pnac117

[21] Fordyce WEIE. Behavioral concepts in chronic pain and illness. In: Davidson PO, editor. The behavioral treatment of anxiety, depression, and pain. New York: Brunner/Mazel Press, 1976. pp. 147–88.

[22] Gauthier N Thibault P Sullivan MJL. Individual and relational correlates of pain-related empathic accuracy in spouses of chronic pain patients. Clin J Pain 2008;24:669–77. 18806531 10.1097/AJP.0b013e318173c28f

[23] Gauvin S Smith KB Chamberlain S Pukall C. Communication patterns in women with provoked vestibulodynia and their partners. Psychol Sex 2019;10:369–82.

[24] Greville-Harris M Hempel R Karl A Dieppe P Lynch TR. The power of invalidating communication: receiving invalidating feedback predicts threat-related emotional, physiological, and social responses. J Soc Clin Psychol 2016;35:471–93.

[25] Haun JN Fowler CA Venkatachalam HH Alman AC Ballistrea LM Schneider T Benzinger RC Melillo C Alexander NB Klanchar SA Lapcevic WA Bair MJ Taylor SL Murphy JL French DD. Outcomes of a remotely delivered complementary and integrative health partnered intervention to improve chronic pain and posttraumatic stress disorder symptoms: randomized controlled trial. J Med Int Res 2024;26:e57322. 10.2196/57322 PMC11530734 39422992

[26] Hemphill RC Martire LM Polenick CA Stephens MA. Spouse confidence and physical function among adults with osteoarthritis: the mediating role of spouse responses to pain. Health Psychol 2016;35:1059–68. 27294596 10.1037/hea0000383 PMC5033682

[27] Heyman RE. Rapid marital interaction coding system. In: Kerig PK Baucom DH, editors. Couple observational coding systems. Mahwah, NJ: Lawrence Erlbaum Associate, 2004. p. 67–94.

[28] Huang C Wu Z Sha S Liu C Yang L Jiang P Zhang H Yang C. The dark side of empathy: the role of excessive affective empathy in mental health disorders. Biol Psychiatry 2025;98:404–15. 39793690 10.1016/j.biopsych.2024.12.020

[29] Ickes W. Empathic accuracy. J Personal 1993;61:587–610.

[30] Kool MB van Middendorp H Lumley MA Bijlsma JWJ Geenen R. Social support and invalidation by others contribute uniquely to the understanding of physical and mental health of patients with rheumatic diseases. J Health Psychol 2013;18:86–95. 22363049 10.1177/1359105312436438

[31] Lamm C Decety J Singer T. Meta-analytic evidence for common and distinct neural networks associated with directly experienced pain and empathy for pain. Neuroimage 2011;54:2492–502. 20946964 10.1016/j.neuroimage.2010.10.014

[32] Leonard MT Cano A Johansen AB. Chronic pain in a couples context: a review and integration of theoretical models and empirical evidence. J Pain 2006;7:377–90. 16750794 10.1016/j.jpain.2006.01.442 PMC1890016

[33] Leonard MT Issner JH Cano A Williams AM. Correlates of spousal empathic accuracy for pain-related thoughts and feelings. Clin J Pain 2013;29:324–33. 23462286 10.1097/AJP.0b013e3182527bfd

[34] Leong LE Cano A Johansen AB. Sequential and base rate analysis of emotional validation and invalidation in chronic pain couples: patient gender matters. J Pain 2011;12:1140–8. 21703939 10.1016/j.jpain.2011.04.004

[35] Linehan MM. Cognitive-behavioral treatment of borderline personality disorder. New York: Guilford Press, 1993.

[36] Linton SJ. Intricacies of good communication in the context of pain: does validation reinforce disclosure? PAIN 2015;156:199–200. 25599438 10.1097/01.j.pain.0000460297.25831.67

[37] Lippert T Prager KJ. Daily experiences of intimacy: a study of couples. Pers Relation 2001;8:283–98.

[38] Martire LM Schulz R Helgeson VS Small BJ Saghafi EM. Review and meta-analysis of couple-oriented interventions for chronic illness. Ann Behav Med 2010;40:325–42. 20697859 10.1007/s12160-010-9216-2 PMC4101802

[39] Martire LM Stephens MA Mogle J Schulz R Brach J Keefe FJ. Daily spousal influence on physical activity in knee osteoarthritis. Ann Behav Med 2013;45:213–23. 23161472 10.1007/s12160-012-9442-x PMC3594506

[40] Martire LM Zhaoyang R Marini CM Nah S Darnall BD. Daily and bidirectional linkages between pain catastrophizing and spouse responses. PAIN 2019;160:2841–7. 31408052 10.1097/j.pain.0000000000001673 PMC6856428

[41] Newton-John TR. How significant is the significant other in patient coping in chronic pain? Pain Manag 2013;3:485–93. 24654903 10.2217/pmt.13.52

[42] Nicholas M Vlaeyen JWS Rief W Barke A Aziz Q Benoliel R Cohen M Evers S Giamberardino MA Goebel A Korwisi B Perrot S Svensson P Wang SJ Treede RD, IASP Taskforce for the Classification of Chronic Pain. The IASP classification of chronic pain for ICD-11: chronic primary pain. PAIN 2019;160:28–37. 30586068 10.1097/j.pain.0000000000001390

[43] Nicola M Correia H Ditchburn G Drummond PD. Defining pain-validation: the importance of validation in reducing the stresses of chronic pain. Front Pain Res (Lausanne) 2022;3:884335. 36313220 10.3389/fpain.2022.884335 PMC9614309

[44] Nudelman G Otto K. The development of a new generic risk-of-bias measure for systematic reviews of surveys. Methodology 2020;16:278–98.

[45] OBrien TB DeLongis A. The interactional context of problem-emotion-and relationship-focused coping: the role of the big five personality factors. J Personal 1996;64:775–813. 10.1111/j.1467-6494.1996.tb00944.x 8956513

[46] Ouzzani M Hammady H Fedorowicz Z Elmagarmid A. Rayyan—a web and mobile app for systematic reviews. Syst Rev 2016;5:210. 27919275 10.1186/s13643-016-0384-4 PMC5139140

[47] Page MJ McKenzie JE Bossuyt PM Boutron I Hoffmann TC Mulrow CD Shamseer L Tetzlaff JM Akl EA Brennan SE Chou R Glanville J Grimshaw JM Hrobjartsson A Lalu MM Li T Loder EW Mayo-Wilson E McDonald S McGuinness LA Stewart LA Thomas J Tricco AC Welch VA Whiting P Moher D. The PRISMA 2020 statement: an updated guideline for reporting systematic reviews. BMJ 2021;372:n71. 33782057 10.1136/bmj.n71 PMC8005924

[48] Pitcher MH Von Korff M Bushnell MC Porter L. Prevalence and profile of high-impact chronic pain in the United States. J Pain 2019;20:146–60. 30096445 10.1016/j.jpain.2018.07.006 PMC8822465

[49] Pollerhoff L Stietz J Depow GJ Inzlicht M Kanske P Li SC Reiter AMF. Investigating adult age differences in real-life empathy, prosociality, and well-being using experience sampling. Sci Rep 2022;12:3450. 35236872 10.1038/s41598-022-06620-x PMC8891267

[50] Preckel K Kanske P Singer T. On the interaction of social affect and cognition: empathy, compassion and theory of mind. Curr Opin Behav Sci 2018;19:1–6.

[51] Quesque F Apperly I Baillargeon R Baron-Cohen S Becchio C Bekkering H Bernstein D Bertoux M Bird G Bukowski H Burgmer P Carruthers P Catmur C Dziobek I Epley N Erle TM Frith C Frith U Galang CM Gallese V Grynberg D Happe F Hirai M Hodges SD Kanske P Kret M Lamm C Nandrino JL Obhi S Olderbak S Perner J Rossetti Y Schneider D Schurz M Schuwerk T Sebanz N Shamay-Tsoory S Silani G Spaulding S Todd AR Westra E Zahavi D Brass M. Defining key concepts for mental state attribution. Commun Psychol 2024;2:29. 39242813 10.1038/s44271-024-00077-6 PMC11332223

[52] Reis HT Shaver P. Intimacy as an interpersonal process. In: Duck S Hay DF Hobfoll SE Ickes W Montgomery BM, eds. Handbook of personal relationships: Theory, research and interventions. Chichester, UK: John Wiley & Sons; 1988:367–89.

[53] Rosen NO Bois K Mayrand MH Vannier S Bergeron S. Observed and perceived disclosure and empathy are associated with better relationship adjustment and quality of life in couples coping with vulvodynia. Arch Sex Behav 2016;45:1945–56. 27164894 10.1007/s10508-016-0739-x

[54] Scheffer JA Cameron CD Inzlicht M. Caring is costly: people avoid the cognitive work of compassion. J Exp Psychol Gen 2022;151:172–96. 34410802 10.1037/xge0001073

[55] Schurz M Radua J Tholen MG Maliske L Margulies DS Mars RB Sallet J Kanske P. Toward a hierarchical model of social cognition: a neuroimaging meta-analysis and integrative review of empathy and theory of mind. Psychol Bull 2021;147:293–327. 33151703 10.1037/bul0000303

[56] Simpson JA Oriña MM Ickes W. When accuracy hurts, and when it helps: a test of the empathic accuracy model in marital interactions. J Personal Soc Psychol 2003;85:881–93. 10.1037/0022-3514.85.5.881 14599251

[57] Singer T Seymour B O'Doherty J Kaube H Dolan RJ Frith CD. Empathy for pain involves the affective but not sensory components of pain. Science 2004;303:1157–62. 14976305 10.1126/science.1093535

[58] Slavich GM. Social safety theory: a biologically based evolutionary perspective on life stress, health, and behavior. Annu Rev Clin Psychol 2020;16:265–95. 32141764 10.1146/annurev-clinpsy-032816-045159 PMC7213777

[59] Stephens MAP Martire LM Cremeans-Smith JK Druley JA Wojno WC. Older women with osteoarthritis and their caregiving husbands: effects of pain and pain expression on husbands' well-being and support. Rehabil Psychol 2006;51:3–12.

[60] Stephenson E Delongis A Esdaile JM Lehman AJ. Depressive symptoms and rheumatoid arthritis: spouse empathic responding as a buffer. Arthritis Care Res 2014;66:532–41. 10.1002/acr.22161 24023009

[61] Treede RD Rief W Barke A Aziz Q Bennett MI Benoliel R Cohen M Evers S Finnerup NB First MB Giamberardino MA Kaasa S Korwisi B Kosek E Lavand'homme P Nicholas M Perrot S Scholz J Schug S Smith BH Svensson P Vlaeyen JWS Wang SJ. Chronic pain as a symptom or a disease: the IASP classification of chronic pain for the international classification of diseases (ICD-11). PAIN 2019;160:19–27. 30586067 10.1097/j.pain.0000000000001384

[62] Vangronsveld KL Linton SJ. The effect of validating and invalidating communication on satisfaction, pain and affect in nurses suffering from low back pain during a semi-structured interview. Eur J Pain 2012;16:239–46. 22323376 10.1016/j.ejpain.2011.07.009

[63] Wallwork SB Shenk C McMurtry CM Hood AM Pavlova M Olson AE Moseley GL Noel M. “I hear you” Validation in the context of children's pain as an untapped opportunity to prevent chronic pain. PAIN 2024;165:2667–72. 39073392 10.1097/j.pain.0000000000003350 PMC11634643

[64] Weiß M Jachnik A Lampe EC Gründahl M Harnik M Sommer C Rittner HL Hein G. Differential effects of everyday-life social support on chronic pain. BMC Neurol 2024;24:301. 39198777 10.1186/s12883-024-03792-z PMC11351827

[65] Wilson SJ Martire LM Sliwinski MJ. Daily spousal responsiveness predicts longer-term trajectories of patients' physical function. Psychol Sci 2017;28:786–97. 28459650 10.1177/0956797617697444 PMC5467745

[66] Ybema JF Kuijer RG Hagedoorn M Buunk BP. Caregiver burnout among intimate partners of patients with a severe illness: an equity perspective. Pers Relation 2002;9:73–88.

